# Systems Biology to Understand and Regulate Human Retroviral Proinflammatory Response

**DOI:** 10.3389/fimmu.2021.736349

**Published:** 2021-11-16

**Authors:** Mohamed Helmy, Kumar Selvarajoo

**Affiliations:** ^1^ Bioinformatics Institute (BII), Agency for Science, Technology and Research (ASTAR), Singapore, Singapore; ^2^ Department of Computer Science, Lakehead University, Thunder Bay, ON, Canada; ^3^ Singapore Institute of Food and Biotechnology Innovation (SIFBI), Agency for Science, Technology and Research (ASTAR), Singapore, Singapore; ^4^ Synthetic Biology Translational Research Program & SynCTI, Yong Loo Lin School of Medicine, National University of Singapore (NUS), Kent Ridge, Singapore

**Keywords:** human retroviral, systems biology, proinflammatory response, computational modeling, cancer

## Abstract

The majority of human genome are non-coding genes. Recent research have revealed that about half of these genome sequences make up of transposable elements (TEs). A branch of these belong to the endogenous retroviruses (ERVs), which are germline viral infection that occurred over millions of years ago. They are generally harmless as evolutionary mutations have made them unable to produce viral agents and are mostly epigenetically silenced. Nevertheless, ERVs are able to express by still unknown mechanisms and recent evidences have shown links between ERVs and major proinflammatory diseases and cancers. The major challenge is to elucidate a detailed mechanistic understanding between them, so that novel therapeutic approaches can be explored. Here, we provide a brief overview of TEs, human ERVs and their links to microbiome, innate immune response, proinflammatory diseases and cancer. Finally, we recommend the employment of systems biology approaches for future HERV research.

## Introduction

After the human genome sequencing project, it is now well-known that only a very minor component of the whole genome, that is, only about 1-2% constitute of protein-coding genes (1). The remaining sequences make up the numerous transcriptional and translational regulatory components, such as ribosomal DNA genes, transfer RNA genes, and non-coding DNA sequences. Notably, in mammalian cells, about half of the non-coding DNA sequences are transposable elements (TEs) (2).

TEs, also referred as transposons, were originally discovered in the 1940s by Barbara McClintock while studying the maize genetics. It was shown that within the genome, TEs were able to transiently move across their location through chromosomal breakage mechanisms (3, 4). Although there were some interests in TEs after their discovery, only during the early 2000s that the topic obtained notable recognition through ENCODE, FANTOM and the roadmap epigenomics projects (5–7). These initiatives helped to identify functional elements of the human genome, especially the non-coding regions.

The dynamic property of large-scale TEs found in eukaryotic genome has possibly provided them the advantage to evolve over environmental changes, disease perturbation or evolutionary pressure. However, at the same time, make them prone to viral infection and disease evolution (8, 9). Thus, research into finding the mechanisms and the role of individual TE is an intense current research domain. However, despite the immense efforts to study and characterize these non-coding elements, the bulk of them still remain poorly understood (10).

**Figure 1:**
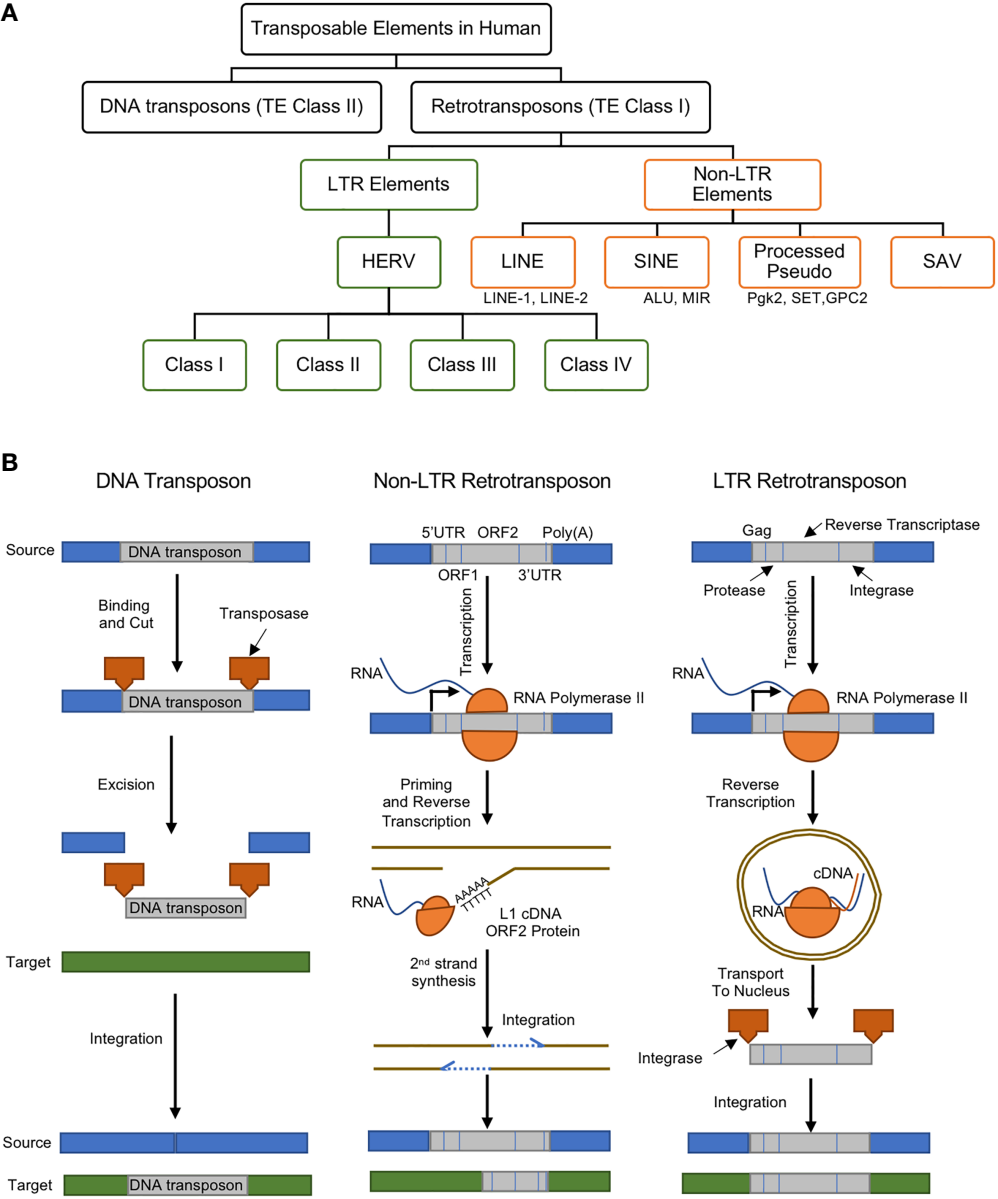
**(A)** Classes and types of transposable elements (TEs) in the human genome, **(B)** gene regulatory mechanisms of DNA transposon (left), non-LTR retrotransposon (middle), and LTR retrotransposon (right). DNA transposons are mobilized through a cut and paste machinery that is based on a transposase that is encoded at their flanked sequences. The transposase cuts the DNA transposon from the source and integrates it into a new genomic location. Retrotransposons require an intermediate step of the reverse transcription of an RNA to be mobilized. Non-LTR retrotransposons are transcribed to produce a full-length mRNA, but its transposition uses target-site primed reverse transcription (TPRT). This produces a single strand of cDNA, which is used to create the complementary strand of the ds-DNA that is integrated in the genomic DNA. This process can lead to target-site duplications (TSDs) and small deletions in the target. Non-LTR retrotransposons encode one or two open reading frames (ORFs). The retrotransposition of the LTR-retrotransposons require the encoding of the Gag protein, protease, reverse transcriptase, and integrase enzymes. The RNA polymerase II of the host recognizes the promoter of the LTR-retrotransposons and creates its mRNA. The Gag makes a virus-like particle that contains the mRNA, the reverse transcriptase and the integrase then uses them to create the full-length double-stranded DNA, which is later integrated into the host’s DNA using the integrase enzyme. Panel **(B)** was modified from (11).

TEs, whose size range anywhere from a few hundred to several thousand pairs of nucleotides, are currently classified into two major categories: i) DNA transposons (or TE Class II) and ii) retrotransposons (or TE Class I) (**Figure 1A**) (12).

## DNA Transposons

DNA transposons are transposase genes that are flanked between two Terminal Inverted Repeat (TIR). They are excised by transposase enzymes and move through a DNA intermediate which recognizes TIRs and become inserted into a new genomic location. Upon insertion and integration, the target site DNA is duplicated (Target Site Duplications), which becomes a unique signature for each DNA transposon (**Figure 1B**, left). The major focus of this manuscript is LTR retrotransposons, particularly the endogenous retroviruses, as will be discussed in better details below.

## Retrotransposons

Retrotransposons comprise of long terminal repeats (LTRs) and non-long terminal repeats (non-LTRs) sequences (**Figure 1B**, middle and right). Retrotransposons are elements that are reverse transcribed into a cDNA and integrated back into a different region of the genome. DNA transposons, on the other hand, are able to insert themselves into different locations with the aid of a circular DNA intermediate (**Figure 1B**) (11). Thus, retrotransposons are considered replicative, while DNA transposons are termed non-replicative. Both types of TEs can be further divided into subclasses, families or superfamilies that share a common genetic organization or a recent ancestral origin (12). Nevertheless, the classification of TEs may most likely evolve as new data or knowledge is generated, and although both retrotransposons and DNA transposons play important gene regulatory roles, there are mounting recent reports showing their associations to about 100 diseases (13).

### Non-LTR Retrotransposons

Non-LTR retrotransposons (also known as polyA retrotransposons or target-primed (TP) retrotransposons), comprises of long and short interspersed nuclear elements (LINEs and SINEs) and they are more like an integrated mRNA (**Figure 1B**, middle) (14). Non-LTR retrotransposons are one of the most abundant retrotransposons in all eukaryotic genomes (15). The estimations show that about 30% of the human genome is directly or indirectly driven by the Non-LTR retrotransposons can due to their excessive copy number (13, 16). LINEs consist of an internal promoter for RNA polymerase II, a 5′ untranslated region (UTR), two open reading frames (ORFs), and a 3′ terminal polyadenylation site (17). SINEs possess an RNA polymerase III internal promoter and a 3′ A-rich tract (17). SINEs partner with LINEs for reverse transcriptase and endonuclease functions. SINE and LINE mutations and polymorphisms have been linked to numerous human diseases (9). For example, increased LINE-1 expression led to the induction of type I interferon in systemic autoimmune disease patients (18).

### LTR Retrotransposons: Retroviruses

The LTR retrotransposons, in eukaryotic genomes, are pairs of identical sequences of DNA that are found on either end of a series of genes, pseudogenes, or endogenous viral elements (**Figure 1B**, right). They are known to comprise roughly a tenth of the human genome (19). In jawed vertebrates and human, the LTR retrotransposons are largely endogenous retroviruses (ERVs).

**Figure 2:**
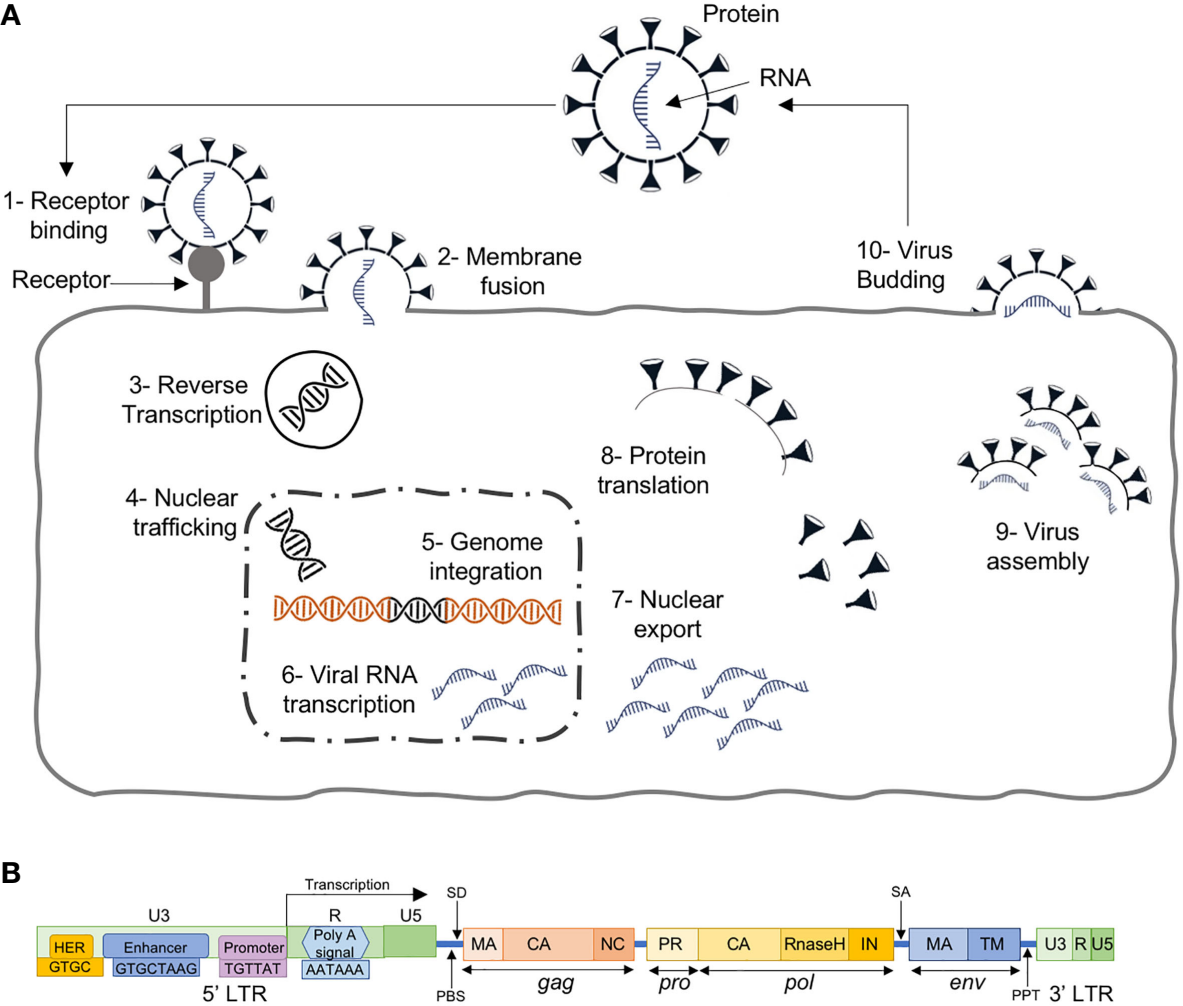
**(A)** The retroviral replication cycle where, after entry into the host, dimeric single stranded RNA (ssRNA) is reverse transcribed into double-stranded DNA (dsDNA) by virion-associated reverse transcriptase enzyme, which subsequently becomes assembled and matures outside the infected cells, **(B)** HERV structure with 4 genes (*gag*, *pro*, *pol* and *env*) between the 5’ and 3’ LTRs.

The ERVs are known to be present in vertebrates for more than 400 million years, and are inherited viral elements from ancient retroviruses that infected germ cells or their progenitors (19, 20). They are able to insert a copy of their RNA genome into the DNA of its host cells’ genome to become a provirus (20). This is done through a few regulatory steps (**Figure 2A**): i) the virus uses “retro” technique in the host cell’s cytoplasm to produce DNA from its RNA genome using its own reverse transcriptase enzyme, ii) transported to the nucleus, the resultant DNA, through an integrase enzyme, is inserted into the host cell’s genome at random, iii) the modified host cell genome transcribes the viral genes, together with its own genes, which eventually leads to multiplication of the original virus.

In their host, these have been inactivated by the accumulation of coding sequence mutations and hence, have reached a so-called genetic fixation through various transcriptional/translational repression and histone hypermethylation. Since the insertions are random, and their functions were initially unknown, these sequences were referred to as junk DNA (21).

HERVs, the ERVs in human, are similar to exogenous retroviruses, consisting of four genes (*gag*, *pro*, *pol* and *env*) between the LTRs (**Figure 2B**). The LTRs serve as promoters with typical RNA regulatory sequences and transcription factor binding sites for HERV protein expression. HERVs are permanently integrated genome elements that are mostly located in the heterochromatin and have undergone methylation silencing (22). That is, after million years of evolution and multiple cycles of integration and reintegration, HERVs have acquired many mutations silencing their activity and remain harmless to the host.

More recently, several HERVs’ roles have been identified, especially for developmental and immune responses in the host (23, 24). The high frequency of HERVs within LTRs indicates that viral adaptations can take advantage of immune signaling pathways that promote viral transcription and replication. They are, thus, still able to regulate and shape the host immune system and may indicate a crucial ancestral role in the evolution and functioning of the mammalian or human immune system. Notably, recent works have shown links between increased HERV protein expressions with human diseases through the proinflammatory pathways, notably in the triggering of interferon responses indicating an involvement in multiple human diseases (25).

## HERVs in Human Diseases

The ability of HERVs to stably integrate into the genome and to modify the expression of nearby genes drew attention for the investigation of their role in different human diseases (26). It was shown that HERVs are highly expressed in neoplastic cells, which indicated a relationship between them and the neoplastic transformation. To date, the relationship between HERVs and several types of cancer is established including soft tissue sarcoma, chronic myeloid leukemia, breast cancer, hepatocellular carcinoma, prostate carcinoma, ovarian carcinoma, and pancreatic cancer (23, 27). HERVs were also linked to neurological disorders (28), mental illness (29), neuropsychological diseases (30), neurodevelopmental disorders and neurodegenerative diseases (31, 32). In addition, they are also found to be involved in Type 1 diabetes (33), autoimmune disorders and multiple sclerosis (34, 35). Here, we will briefly review the relationships between HERVs and microbiome, their involvement in immunity, proinflammatory diseases and cancer.

### HERVs and Microbiome

Commensal microbiota, communities of thousands of microorganisms, are known to play key roles in modulating host immune systems and their homeostasis maintenance (36). Nevertheless, the cellular processes by which microbiota benefits the host cells in still largely unknown.

One recent work studied the effect of skin microbiota on ERVs. Keratinocytes promoted the induction of commensal-specific T cells, attributed by the discrete expression of defined ERVs, which activated cyclic GMP-AMP synthase (cGAS), stimulator of interferon genes protein (STING) signaling. Subsequently, ERV reverse transcription inhibition resulted in aberrated immunity to the microbiota and its associated tissue repair function (37).

Large-scale ERVs expression was investigated by high-throughput microarray in murine and human immune cells (38). Wildtype and MYD88 knock out (KO) mice revealed that certain ERVs expression were dependent on gut microbiota. Notably, ERVs expression were largely decreased in MYD88 KO mice, indicating that microbiota is necessary to induce ERVs expression through MYD88 signaling in mice’s gut.

In human, one study demonstrated a strong correlation between the expression of HERV-H, -K and -W, and the concentration of *Bifidobacterium* spp., indicating that the colonization with commensal microbes made up of *Bifidobacterium* spp. causes global modulation of HERVs in the gut (39).

Thus, these works provide evidence that ERVs can regulate immune response through microbiome, and, collectively, they may be better controlled for health benefits.

### HERVs in Innate Immunity

HERVs have become an integral part of host immunity and are able to protect the host from exogenous retroviral infections. HERV components can be detected by several innate immunity sensors, such as the pattern recognition receptors (PRRs), which play pivotal roles in antiviral protections.

**Figure 3:**
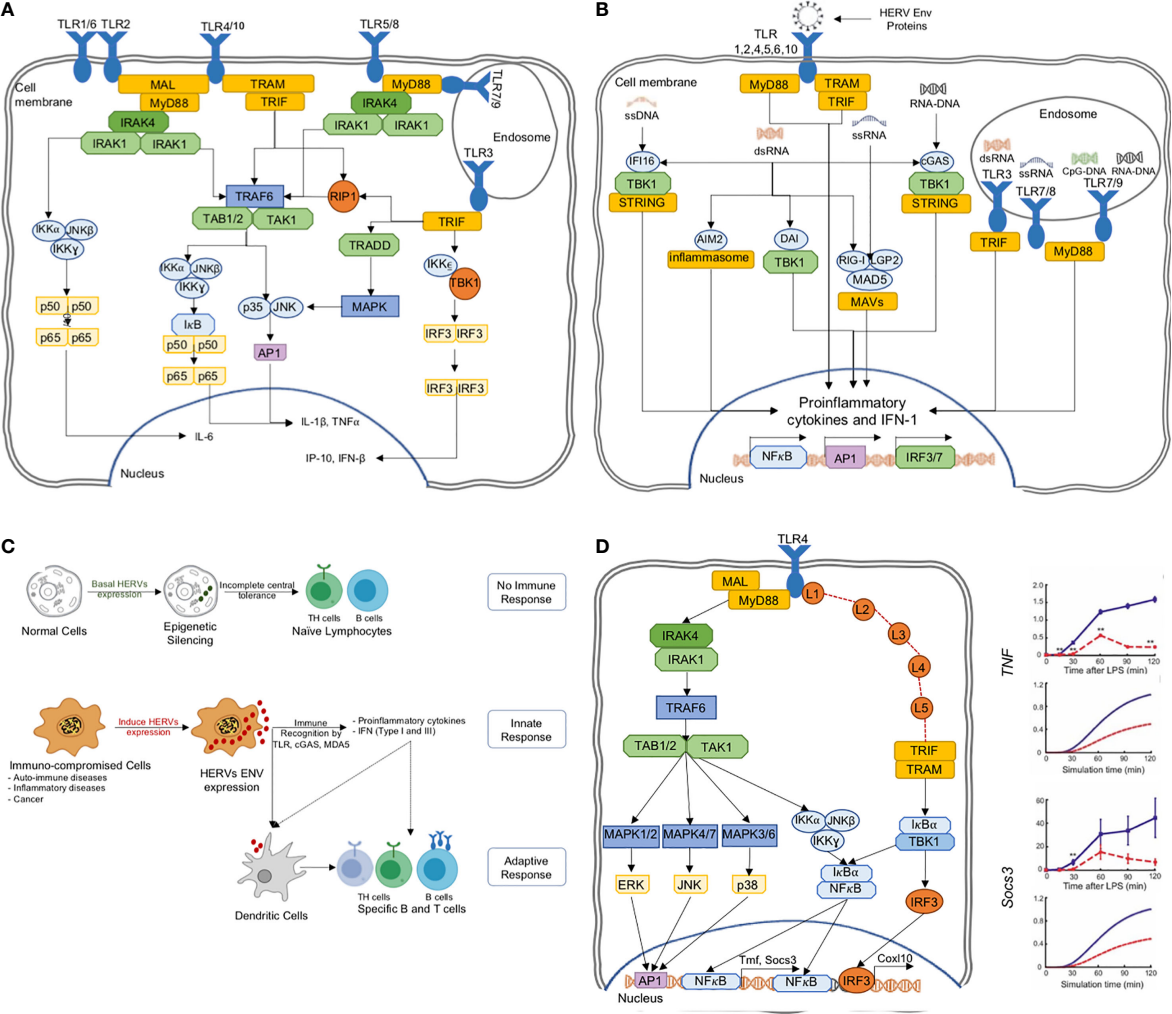
**(A)** The TLR pathways in human triggering the proinflammatory signal transduction, **(B)** TLRs are able to recognize several HERV ENV proteins to activate the proinflammatory responses, **(C)** HERV ENV proteins are able to stimulate adaptive immune response through T cell differentiation and antigen presentation, **(D)** TLR models can predict experimentally verifiable novel intermediates, which could be targeted for controlled proinflammatory regulation (42–45).

A major class of PRRs are the Toll-like receptors (TLRs). There are ten types found in humans, and they play the first line of defense in detecting a wide variety of pathogenic interventions (40, 41) (**Figure 3A**). In particular, TLR3,7,8 and 9 are endosomal and together they sense dsRNA, ssRNA and CpG DNA. At plasma membrane, the TLR2/6/10 are known to detect HIV viral proteins such as GAG (p17, p24) and ENV (gp41, gp120). On the other hand, TLR4 senses ENV, HIV-TAT and other virion-associated bacterial components such as lipopolysaccharide (LPS) (46). These diverse TLR engagements with viral proteins or components lead to the activation of MAP kinases and NF-κB and the resultant proinflammatory cytokine production for neutralization of the infections (23) (**Figure 3B**). Moreover, ENV stimulates dendritic cells to promote T helper cell differentiation (**Figure 3C**).

The HERV-W Env was shown to interact with TLR4 to induce cytokines IL-1, IL-6 and TNF-α (47). In another example, HERV-K(HML-2) Env activated TLR7/8 in neurons and microglia, and strongly correlates with the onset and causing neurodegeneration *via* proinflammatory response (28, 48). Similarly, HERV-H was shown to be associated with multiple sclerosis (49). HERV-derived peptides, involving ENV conserved subunit ISD, have been implicated in immune-suppressive mechanisms (35). On the other hand, during pregnancy, the HERV protein, Syncytin-2, immunosuppresses T cells in an exosome-mediated manner (50). Thus, there are mounting evidence pointing to the importance of HERVs in triggering the human innate immune response. While some of the HERV sequences activate the immune system, others suppress it. Thus, a deeper knowledge of TLR and HERV interactions are necessary for the positive regulation of proinflammatory response for therapeutic agents.

In a transcriptome-wide RNA-Seq analysis of murine macrophages, full-length ERV-derived lncRNA (lnc-EPAV) expression was rapidly upregulated by viral RNA mimics or RNA viruses (2). Silencing lnc-EPAV showed that it facilitated the expression of RELA, an NF-κB subunit that plays a pivotal role in innate immunity (51). lnc-EPAV-deficiency also reduced the expression of type I interferons (IFNs). Following lethal RNA virus infection, this resulted in increased viral loads and mortality.

Another transcriptome-wide analysis of B cells revealed a small number of highly distinct ERVs that are strongly and consistently induced during murine B cell activation (52). Notably, a single endogenous MLV provirus, Xmv45, was found to be expressed at significantly higher levels.

Another research area for exploiting HERV is to regulate the tumor suppressor p53 in cancer. By DNA damage and cellular stress, the p53 pathway leads to cellular apoptosis. It was previously found that 30% of binding sites were located within copies of a few HERV families (23, 53). This benefits retroviruses as p53 pathway leads to rapid induction of transcriptional processes that neutralizes viral RNA from the host cell.

Having mentioned that HERV can be positively regulated, it must also be noted that the random insertion mechanisms of retroviruses could unintentionally activate oncogenes or deactivate tumor suppressing genes (54). In other words, HERVs have the potential to transform normal cells into cancerous cells. Moreover, proviruses are known to remain latent for extended period of time within the host and could likely become activated by key changes to host cell environment.

### HERVs in Cancer

Back in the early 70s, there has been studies on reverse transcriptase activity and oncoviruses in human cancers (55, 56). Subsequent works eventually led to the link between HERVs activations and myriad cancers (57). Elevated expression of the oncogene ETV1 through HERV-K_22q11.23 5′-LTR-UTR sequence has been shown in prostate cancers (58). In melanoma, the activation of MEK- ERK and p16INK4A-CDK4 pathways increased the expression of HERV-K (59). There are numerous other links that show the connection or causality between cancer and elevated HERV expressions (60). Such mounting evidences have led to the development of immunotherapeutic strategies that targets HERVs (61, 62). Nevertheless, these methods may not be effective as the detailed mechanistic understanding, such as how the upstream signaling process that activates the downstream distinct HERVs in diverse cancers are still largely unknown. For example, a dynamic computational model describing the detailed signaling reactions, from TRAIL ligand-receptor binding, was used to identify a crucial intracellular target, protein kinase C, whose downregulation sensitized cancer cell death from 60% to 95% *via* increased apoptosis signaling (63). Detailed mechanistic understanding such as tracking intracellular signaling dynamics, thus, can better help to elucidate crucial specific targets that selectively control specific set of downstream proinflammatory or apoptosis responses.

It is noteworthy to mention that TLRs’ expressions are also elevated in many cancers (64, 65). Hence, it is conceivable that HERVs can be expressed *via* TLR signaling, especially that there are evidences for increased NF-κB, MAPK and IRF1 activity with increased HERV expressions (59, 66, 67). Thus, further research into TLR-induced HERV expressions will provide better clues into finding the missing mechanistic links.

### HERVs in Neuro and Autoimmune Diseases

HERVs (also LINEs and SINEs) have been identified in numerous neurodegenerative diseases, including schizophrenia, Alzheimer’s disease, stroke, multiple sclerosis (MS), amyotrophic lateral sclerosis, accelerated neurological decline in aging, and neuropathogenesis of severe acute respiratory syndrome coronavirus-2 (SARS-CoV-2) (9, 68)

In MS, HERV-W env binds with TLR4 on oligodendroglial precursor cells and induces pro-inflammatory response leading to the suppression of myelin expression in MS lesions (69). HERV-W env is also known to be a superantigen linked with demyelination in MS and, consequently, HERV-W env antibody have been shown to effectively rescue myelin expression (47, 70).

High-throughput RNA-Seq was performed to investigate the expression and role of HERVs in systemic lupus erythematosus (SLE) (71). Between control and test case datasets, 481 HERV encoding regions were identified, and significant variations between differentially expressed HERVs and non-differentially expressed HERVs were determined. Notably, HERV-K, HERV-H, and MER4 were over-expressed in SLE patients. Subsequently, utilizing locus specific resolution of HERV mapping, key innate immune pathways regulated by the differential HERV expression in SLE were identified.

So far, we have introduced our basic understanding of TEs, HERVs, the innate immune signaling through TLRs, and the causal relationship between HERVs and proinflammatory diseases and cancers. Although we are gaining a better understanding on the retrovirus effects on host proinflammatory response and their role in cancer, there is no simple way to generalize the behaviors under different conditions and cell types. As noted, in some cases, the HERVs are able to activate immunity while in others it de-activates. To tackle the conundrum, we can explore computational models that are able to aid in the distinction of differing proinflammatory kinetics.

## Systems Biology and Machine Learning

Computational modelling is becoming an integral part of biological research, especially when investigating cellular network responses. There are a wide range of computational modeling techniques and algorithms today that provide novel mechanistic insights (72). The methods can be broadly classified into i) parametric approaches such as dynamic modeling using ordinary differential equations (42, 63, 73–75), and ii) non-parametric models using Boolean logics, stoichiometric matrix and Bayesian inference algorithms (76, 77).

Dynamic models are mostly built using differential equations to represent each step in a cellular network, such as signaling or metabolic reaction. Using the reaction topology and kinetics, the models predict dynamic outcomes to different *in silico* perturbations, or to understand the key regulatory mechanisms, such as bottlenecks, and flux distributions (78, 79). In other words, the dynamic models utilize *a priori* knowledge of cellular networks and temporal experimental data to simulate the molecular activation or concentration profiles over time.

Although dynamic or kinetic models have been widely used and have proven their benefits, stoichiometric constraint-based modeling and Boolean logic-based approaches are also popular. These models are used when reactions kinetics are not feasible to obtain and will largely provide qualitative information on the outcome of any perturbation that need to be modeled or tested (80).

In a previous study, using a perturbation-response kinetic modeling approach, we simulated the differential dynamic activation kinetics of NF-κB and MAP kinases in MyD88 and TRIF mutant cells to TLR4 activation (43). Key predictions of the model include the enhancement of alternative pathways at pathway junctions through signaling flux redistribution, and the presence of novel intermediates on TRIF-dependent pathways, which were subsequently verified experimentally and characterized (42, 44, 45) (**Figure 3D**). Similar successes were also noted for TLR3, TNF and TRAIL signaling, where dynamic models were able to elucidate novel mechanistic features of signaling processes (63, 73–75).

In another study, Hoffmann and colleagues investigated the cell type differential activation mechanisms of IKK–IκB–NF-κB signaling module to TNF and TLR4 stimulation in murine embryonic fibroblasts (81). Their ordinary differential equations model predicted the rapid termination of IKK activity in TNF stimulation is by the negative feedback control through post induction of A20, whereas the prolonged IKK activation in LPS stimulation is caused by positive autocrine feedback of TNF signaling.

For cancer, mathematical modeling of signaling pathways was also used to investigate different aspects of tumor growth and progression. Hendrata and Sudiono presented a model based on partial differential equations to study apoptosis in HeLa cancer cells (82). The model simulations were fitted with experimentally profiles observed tumor growth rate and phenotypic patterns, and this was subsequently used to predict the growth of tumours over time for various mesenchymal stem secretions. There are also numerous other cancer and proinflammatory signaling models that have provided indispensable data for guiding experimental elucidation of key regulatory properties (83–86). Thus, computational and systems biology research could aid in elucidating the regulatory properties of diverse HERVs provided time-series experiments are performed in wildtype and mutant experiments. However, traditional computational modeling approaches, both parametric and non-parametric, have limitations in their capabilities, scale or required data (87).

Machine learning approaches were used in combination with systems biology to make prediction models where traditional modeling approaches failed due to their limitations or the unavailability of sufficient data. This was demonstrated in different fields including cancer, metabolic engineering and proinflammatory disease (87–89). In metabolic engineering, systems biology approaches combined with machine learning models were able to overcome several limitations in data quality and modeling scale (87). Machine learning models in proinflammatory disease helped to establish a relationship between the peptides amino acid sequence (the epitope of the antigen) and the induced proinflammatory response, an approach that can be adopted in the HERV research to investigate the immune response to HERVs (88). Machine learning also solved the challenge of predicting cancer survival and its associated pathways from gene expression data and predicted the survival and association in four types of cancer (89).

Unlike current signaling or metabolic network modeling developed and used, HERV modeling is faced with limited or sparse information that make it a big challenge to even start constructing a workable model. Different computational models require different types of data and levels of quality that help those models perform to the best of their capabilities. For instance, dynamic models require detailed information on the signaling pathway, its reactions, the rate of the reactions, gene expressions and multiple time points (87). Furthermore, other types of data (such as genomic and protein sequences and 3-D structure data) are important for predicting molecular interactions and signaling pathways associations (90). Such data is not readily available for HERVs as they are understudied (10). To overcome this limitation, systems biology coupled with machine or deep learning models provide hope for further understanding the roles of HERVs on immune response or cancer progression. This approach could provide valuable clues for predicting causal functions using heuristics methods (91).

For example, most mammalian retroviruses envelope glycoproteins are linked to group-specific antigen (gag) genes, which codes for matrix proteins (MAs) involved in virus component assembly, transport and budding. Notably, HERV gag protein have been shown to correlate with prostate cancer progression (92). Since, the initiation sites and termination sites of gag gene transcription are poorly understood, Ma *et al.* developed a computational method using support vector machine and random forest to identify MAs in HERVs and predict the initiation sites and termination sites. Their model scanned 94,671 HERV sequences from 118 families to predict 104 new putative MAs in human chromosomes (93).

Using a similar approach, Dey et al. investigated the protein-protein interactions between human and SARS-CoV-2 virus. Using different sequence-based features of human proteins, e.g. amino acid composition, pseudo amino acid composition, and conjoint triad, they predicted 1,326 human target proteins of SARS-CoV-2 and compared them with gene ontology and KEGG pathway enrichment (94). In a more recent work, Kojima et al. used machine learning methods, using *k*-mer occurrence of ancient RNA viral sequences, to identify novel viruses in the human genome (95). Thus, although new in application, machine learning techniques will play a bigger role in elucidating the sequence and regulatory causality between HERVs and major diseases, and will aid in better understanding of the complex interplay between HERVs, human proinflammatory response and diseases like cancer, multiple sclerosis (MS) and AIDS.

## Conclusion

HERVs are TEs that make up a large portion of the human genome. Although mostly underexplored, many of them are known to be activated during embryogenesis and are epigenetically silenced subsequently (96), while others are known to cause host genome instability leading to major diseases such as inflammation and cancer (23, 25). During evolution, HERV genome sequences have acquired large scale mutations and deletions and, therefore, are unable to produce infectious viral agents.

HERV can be transcriptionally activated and are recently known to regulate proinflammatory responses, nevertheless, the details of the regulation remain largely a mystery. Understanding the role of each HERV in general cellular functions such as growth, cell division, differentiation, and immune responses are vital to reveal the connection of HERVs in human diseases. For example, with the ability to regulate innate response through TLRs and the resultant adaptive immune response through antigens recognition, they could be explored as future agents for cancer immunotherapy (62, 97). Furthermore, HERV expressions are mostly silenced by DNA methylation and histone modification. Epigenetic regulatory drugs have, thus, been indicated to reactivate HERVs for immune related clearance (22, 98).

Overall, recent works on HERVs suggest that they could be positively tuned to fight major human diseases. In the near future, we will likely witness the extension of systems biology, through computational modeling of regulatory pathways, and machine learning techniques, through heuristic modeling and data analytics, to help support in overcoming the difficulty of putting a wealth of HERV information together into a hypothesis driven solution to tackle and treat complex diseases.

## Data Availability Statement

The original contributions presented in the study are included in the article/supplementary material. Further inquiries can be directed to the corresponding author.

## Author Contributions

MH wrote the manuscript and prepared the figures. KS wrote the manuscript and led the project. All authors contributed to the article and approved the submitted version.

## Conflict of Interest

The authors declare that the research was conducted in the absence of any commercial or financial relationships that could be construed as a potential conflict of interest.

## Publisher’s Note

All claims expressed in this article are solely those of the authors and do not necessarily represent those of their affiliated organizations, or those of the publisher, the editors and the reviewers. Any product that may be evaluated in this article, or claim that may be made by its manufacturer, is not guaranteed or endorsed by the publisher.
